# Hespi: a pipeline for automatically detecting information from herbarium specimen sheets

**DOI:** 10.1093/biosci/biaf042

**Published:** 2025-07-17

**Authors:** Robert Turnbull, Emily Fitzgerald, Karen M Thompson, Joanne L Birch

**Affiliations:** Melbourne Data Analytics Platform; Melbourne Data Analytics Platform; Melbourne Data Analytics Platform; School of BioSciences at the University of Melbourne, Melbourne, Victoria, Australia

**Keywords:** herbarium, specimen, large language model, optical character recognition, document layout analysis

## Abstract

Specimen-associated biodiversity data are crucial for biological, environmental, and conservation sciences. A rate shift is needed to extract data from specimen images efficiently, moving beyond human-mediated transcription. We developed Hespi (for *herbarium specimen sheet pipeline*) using advanced computer vision techniques to extract authoritative data applicable for a range of research purposes from primary specimen labels on herbarium specimens. Hespi integrates two object detection models: one for detecting the components of the sheet and another for fields on the primary specimen label. It classifies labels as printed, typed, handwritten, or mixed and uses optical character recognition and handwritten text recognition for extraction. The text is then corrected against authoritative taxon databases and refined using a multimodal large language model. Hespi accurately detects and extracts text from specimen sheets across international herbaria, and its modular design allows users to train and integrate custom models.

There are over 3800 active herbaria globally, containing more than 400 million physical specimens (Thiers 2025). Specimen-associated biodiversity data are sought after for biological, environmental, climate, and conservation sciences (Lacey et al. 2017, Davis 2023). Increased investment in natural history collections-based digitization efforts has significantly increased the availability of high-resolution specimen images in the last decade (Walker et al. 2022, Davis 2023). However, transcription rates associated with the digitization of biodiversity data have remained flat (Vollmar et al. 2010, Guralnick et al. 2024a). A rate shift for data extraction from specimen images is required to eliminate this impediment to the mobilization of biodiversity data. Advanced computer vision techniques hold the potential to achieve that rate shift by reducing the time required for digitization of text-based label data and, in doing so, to mobilize vast quantities of biodiversity data from digital specimen images (Walker et al. 2022, Thompson and Birch 2023). Natural language processing methods also have the potential to increase accuracy of text digitization from specimen labels by enabling language detection and terminology extraction (Owen et al. 2020). In this article, we introduce Hespi, a herbarium specimen sheet pipeline. Hespi detects components of specimen sheets, detects the fields in the primary specimen labels and recognizes the text using optical character recognition (OCR), handwritten text recognition (HTR), and multimodal large language models (LLMs).

Herbarium specimens contain a preserved biological sample and both primary (e.g., taxonomic identity, collector, collection date or location) and secondary (e.g., redetermination or confirmation of taxonomic identity, date of curation event) collection data. These specimens provide a verifiable record of the presence of a taxon at a point in time (Funk et al. 2018, Kirchhoff et al. 2018). Historically, specimen-associated data were documented on paper (Groom et al. 2019, Walton et al. 2020), recorded in field notebooks, or transcribed into printed catalogs, and primary and secondary data were written on labels that were attached to the specimens. Mobilization of these specimen data is typically achieved by processing specimens through a digitization workflow, involving the production of a digital specimen image followed by the extraction of text data from that digital image either manually (i.e., via a human intermediary) or semiautomatically (Nelson et al. 2015, Kirchhoff et al. 2018, Hidalga et al. 2020, Thompson and Birch 2023, Guralnick et al. 2024b). Their digitization, conforming to biological data standards (e.g., Access to Biological Collections Data and DarwinCore; Holetschek et al. 2012 and Darwin Core Maintenance Group 2023, respectively), is essential for ensuring their availability for reuse (Groom et al. 2019). The conversion of imaged labels into digital text and the parsing of that text into standard data fields are some of the slowest steps in the digitization pipeline and are significant bottlenecks for biodiversity data mobilization (Tulig et al. 2012, Kirchhoff et al. 2018, Groom et al. 2019, Walton et al. 2020, Weaver et al. 2023, Guralnick et al. 2024a).

Deep learning models using artificial neural networks have been shown to be effective for data extraction from digital images, including phenological (Pearson et al. 2020, Mora-Cross et al. 2022) or morphological (Mora-Cross et al. 2022, Wilson et al. 2023) data, taxonomic identifications (Shirai et al. 2022, Joly et al. 2024), and other textual data (Walker et al. 2022, Milleville et al. 2023, Guralnick et al. 2024a, Weaver 2024, https://github.com/Gene-Weaver/VoucherVision.git). Such techniques hold potential to reduce the reliance on human-mediated transcription and processing of specimen data (Owen et al. 2020, Milleville et al. 2023). Neural network models require large data sets of carefully curated and labeled images for training, to create models that achieve good performance (Walker et al. 2022). The optimal techniques and parameters for tasks required for data extraction from herbarium specimens continue to be elucidated. We have previously described training a deep learning model to detect the various components of a specimen sheet (Thompson et al. 2023a). In the present article, we extend this approach as part of a larger pipeline to extract textual information from primary specimen labels to enable their digitization.

OCR protocols have long been recognized as holding potential for mobilization of textual data from specimens. However, this potential has not yet been realized because of limitations in the accuracy of data extraction using OCR software. Workflows have progressed from applying OCR to whole specimen sheets (Haston et al. 2012, Tulig et al. 2012, Drinkwater et al. 2014) to (manually) identifying the label area and applying OCR (Anglin et al. 2013, Barber et al. 2013, Alzuru et al. 2016, Haston et al. 2017, Dillen et al. 2019). It has been demonstrated that applying OCR to the label-only image is more effective than applying OCR to the whole image (Alzuru et al. 2016, Haston et al. 201^7^, David et al. 2019), and that running OCR over individual text lines cropped from a label image is faster than processing the whole label (David et al. 2019).

The capture of handwritten text is one of the most challenging aspects of OCR. Handwritten text does not always conform to standard character shapes or sizes, which poses a significant challenge for OCR (Owen et al. 2020). The text on natural history specimen labels provides additional challenges, because labels may contain a mixture of handwritten and typed text or the handwriting of multiple individuals (Owen et al. 2020). Tests of accuracy for software targeted at HTR (e.g., ABBYY Fine Reader Engine, Google Cloud Vision) indicate that HTR tools can capture a proportion of specimen data that is accurate and of high quality suggesting that this technology is already a viable technique for data capture (Haston et al. 2017, Owen et al. 2020).

LLMs hold potential to work with unstructured text extracted from specimen labels to both parse and accurately format data to meet curatorial requirements (Weaver et al. 2023, Guralnick et al. 2024b). LLMs are trained to predict the next token in a textual sequence. This self-supervised task allows for massive data sets to be used in training and produces models that are able to perform sophisticated tasks in natural language processing. Multimodal LLMs allow nontextual data inputs such as images and combine these sources to handle complex data processing tasks. The application of multimodal LLMs to the extraction of text from specimen sheets has been discussed by Weaver and colleagues (2023) and Guralnick and colleagues (2024a).

## Applying machine learning tools for the extraction of specimen data

**Figure 1.**
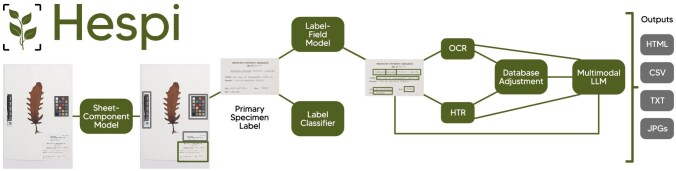
The Hespi pipeline. Specimen sheet available at https://online.herbarium.unimelb.edu.au/collectionobject/MELUA118997a.

A diagram of the Hespi pipeline is shown in figure 1. The stages of the pipeline are explained in more detail below. Briefly, Hespi first takes a specimen sheet and detects the various components within it using the sheet-component model. The primary specimen label (defined below) is detected and is cropped to serve as input for the label-field model, which then detects text in a subset of data fields written on the label. A neural network label classifier is used to determine the type of text (typeset or handwritten) on the label. The text within each field is recognized using OCR and HTR engines. The recognized text is postprocessed and cross-checked against authoritative plant and fungal name lists for specific fields. The primary specimen label and the recognized text are given to a multimodal LLM for correction. The final extracted labels and text data are written to an HTML report and a CSV file for viewing and subsequent data processing.

### The sheet-component model

This object detection model is based on our previous work (described in Thompson et al. 2023a). It takes specimen sheet images and outputs bounding boxes for 11 components:

1. the primary specimen label, containing the data recorded at the time of collection of the plant material or that is provided with the specimen for accession into a herbarium (referred to as the *institutional label* in Thomson et al. 2023)
2. data on the specimen sheet outside of a label (may include primary collecting data or curatorial data, often handwritten)
3. taxonomic and other annotation labels, including taxonomic or curatorial annotations
4. herbarium stamps
5. swing tags attached to specimens
6. the number placed outside the primary specimen label (e.g., herbarium accession number, collector's collection number, donation or loan number)
7. small database labels, printed from collection management system for provision of unique specimen identifier
8. medium database labels, printed from collection management system containing unique specimen identifier and additional taxonomic or curatorial data (e.g., taxonomic name)
9. full database labels, printed from collection management system where primary collection or curation data are born digital (not shown)
10. color target
11. scale.

**Figure 2.**
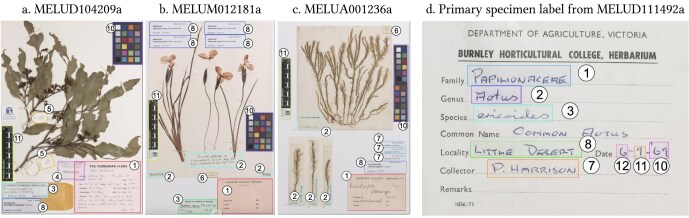
Examples of the prediction classes for the sheet-component model (a–c) and the label-field model (d). The numbers correspond to the listing of classes in the respective sections. The specimen sheets are available at University of Melbourne Herbarium website.

Examples of these components are shown in figure 2a–2c. A data set with annotations corresponding to these components is publicly available on FigShare (Thompson et al. 2023b). It includes 4821 specimen sheet images annotated with bounding boxes for the various components (see the “Sheet-component model” section above), of which 1180 are designated for validation. Of these, 4371 come from The University of Melbourne Herbarium (MELU) and 50 images come from each of the following nine herbaria from the benchmark data set by Dillen and colleagues (2019): Meise Botanic Garden; Royal Botanic Gardens, Kew; The Natural History Museum, London; ZE Botanischer Garten und Botanisches Museum, Freie Universität Berlin; Royal Botanic Garden Edinburgh; Muséum National d'Histoire Naturelle, Paris; University of Tartu; Naturalis Biodiversity Center; and University of Helsinki.

**Figure 3.**
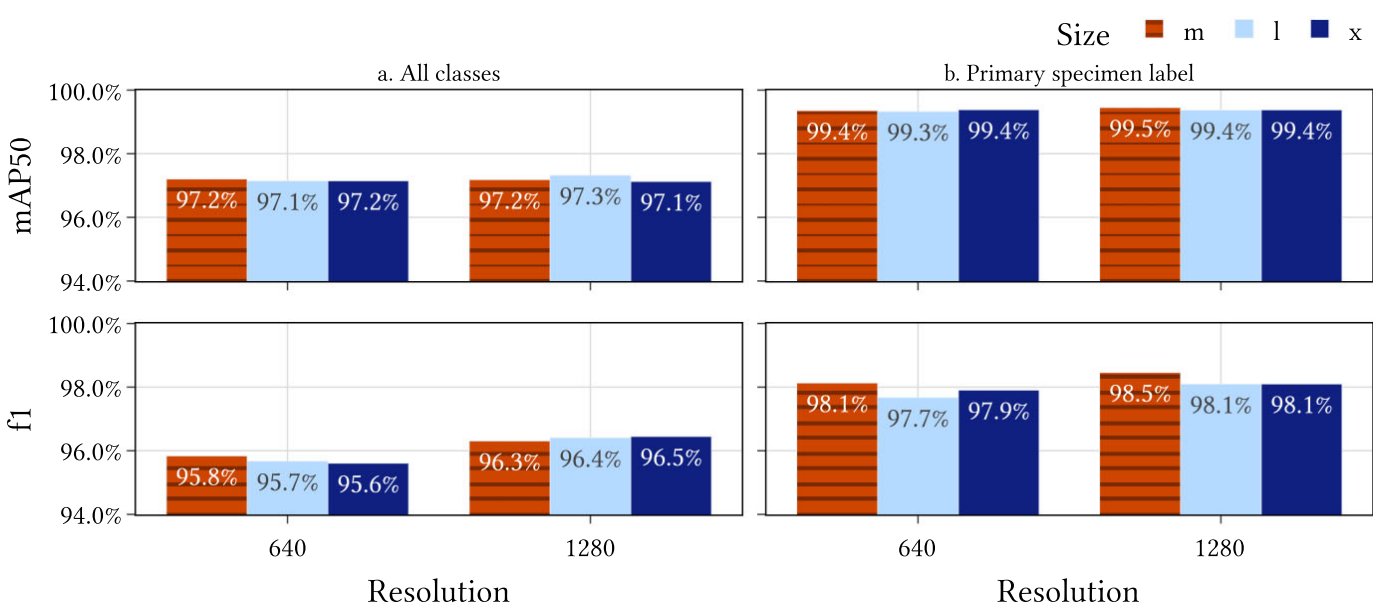
Validation results for YOLOv8 models trained on the sheet-component annotations. The plots on the left (a) show the results for all component classes and the plots on the right (b) show the results for just the primary specimen label.

Thompson and colleagues (2023a) discussed the results for training a version of this model using YOLOv5 (for *you only look once*; Jocher et al. 2020). YOLO is a neural network object detection model that simultaneously predicts bounding boxes and class probabilities. Thompson and colleagues (2023a) trained a YOLOv5 model on the MELU annotations and then fine-tuned on the annotations for the other nine herbaria. In the present article, we use YOLOv8 (Jocher et al. 2023) and train using all the annotations together. Six versions of the model were trained, with resolutions at 640 and 1240 pixels and sizes medium (m), large (l) and extra-large (x). The mean average precision at an intersection over union value of 50% (mAP50) and the f1 score on the validation set for each of these models is calculated (figure 3). For the label-field model, the most critical component is the primary specimen label (elsewhere referred to as *herbarium, specimen, collection, institutional*, and *original* label). The primary specimen label typically contains information about the specimen that needs to be digitized and this is the component used for downstream tasks in the pipeline. The highest f1 score for accurate detection of the primary specimen label was 98.5%, which was achieved with a model size of x and a resolution of 1280 is used in the Hespi pipeline as that configuration provided optimal detection of the primary specimen label component (highest f1 score = 98.5%).

### The label-field model

The label-field model takes any primary specimen label detected from the sheet-component model and detects bounding boxes for the following fields:

1. family
2. genus
3. species (i.e., the specific epithet)
4. infraspecific taxon
5. the authority of the taxon at the lowest rank provided
6. the collector's field number
7. the collector
8. the locality
9. the geolocation (latitude, longitude, elevation, and elevation units)
10. the year
11. the month
12. the day

Examples of these components are shown in figure 2d.

**Figure 4.**
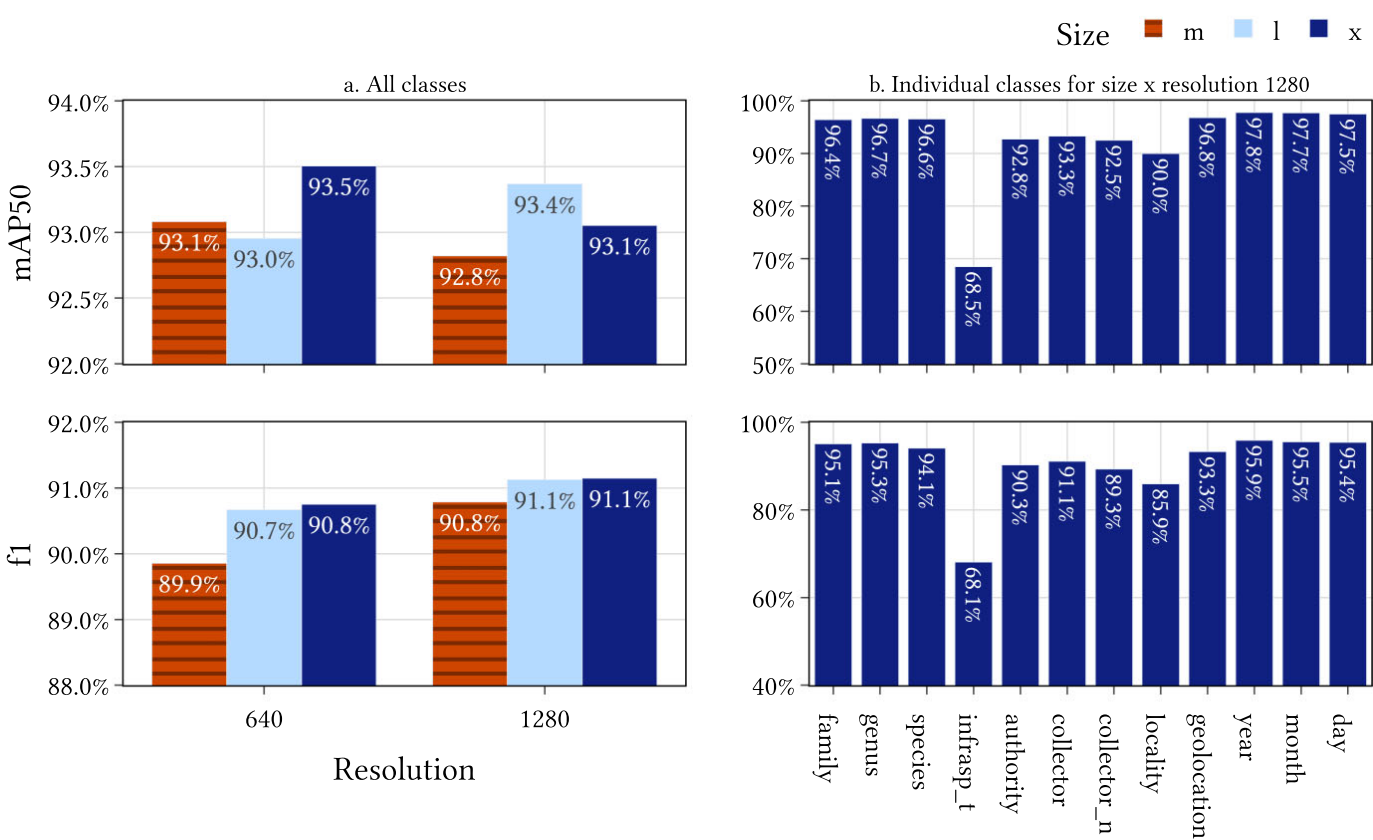
The results on the validation set for the label-field model. The two graphs on the left for all classes (a) show the results for all classes at different resolutions and YOLOv8 model sizes. The two graphs on the right for individual classes (b) show the results for each individual class at a model size of “x” and a resolution of 1280.

These classes were annotated on 3642 images of primary specimen labels from 10 herbaria. A total of 2603 images are from MELU and the remainder are from the nine herbaria represented in the benchmark data set described by Dillen and colleagues (2019). These were broken down into 2887 training images and 755 validation images. The images and annotations are available on FigShare (Turnbull et al. 2024b). The model was trained using YOLOv8 at three different sizes (m, l, and x) and at two resolutions (640 and 1280; figure 4a). The model size x at a resolution of 1280 gave the highest overall f1 score and this is the model configuration that is used in the Hespi pipeline. The results of this model for each field class are shown in figure 4b.

### Label classifier

We have trained a classifier to detect the following types of writing on the primary specimen label: typewritten, printed, handwritten, combination, or empty.

**Figure 5.**
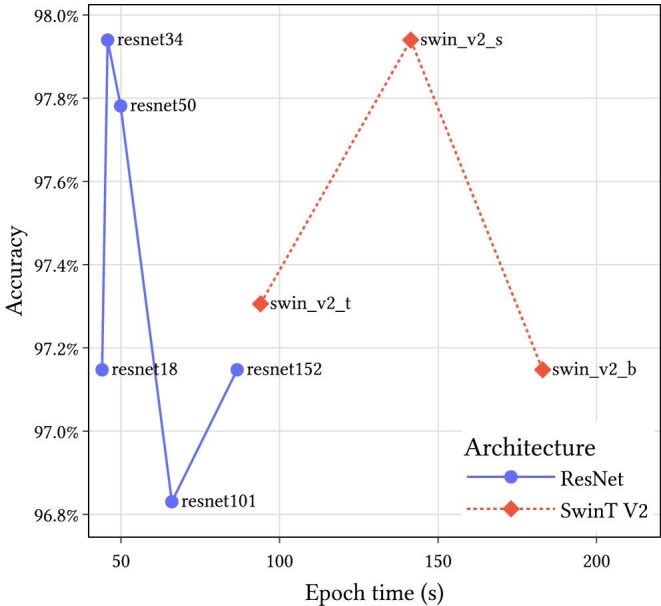
Validation results compared with training time per epoch for the label classifier models.

These writing types were annotated to the 3152 images from the MELU data set. This data set was partitioned into 2521 training images and 631 validation images. Images and annotations are available on FigShare (Turnbull et al. 2024a). Pretrained ResNet (He et al. 2016) and Swin Transformer V2 (Liu et al. 2022) models were used and fine-tuned on this data set using torchapp (Turnbull 2023) for 20 epochs with a batch size of 16 and at a resolution of 1024 pixels. The best-performing models were the ResNet-34 and the Swin Transformer V2 of size s with an accuracy of 97.9% (figure 5). Increasing the model size beyond these configurations led to reduced performance, likely because of overfitting on the training data set. This is potentially due to overfitting to the training data set. The ResNet-34 model is used as part of the Hespi pipeline because of its lower computational complexity.

### Text recognition

Each field detected by the label-field model is input into the text recognition module. This uses the Tesseract OCR engine (Kay 2007, Smith 2007) and the TrOCR large HTR model (Li et al. 2023).

Text formatting is applied to Tesseract or TrOCR results for the family, genus, and the specific epithet fields, where a standardized format is expected. Family and genus fields are changed to title case, specific epithet to lower case. For all three, punctuation marks are stripped from the beginning and end of the text, as well as any whitespace or empty characters.

For the family, genus, specific epithet and authority fields, any recognized text is cross-checked against the World Flora Online database (WFO 2023), an international compendium of vascular plants and mosses, and against databases within the Australian National Species List, a nationally recognized taxonomy of Australian biodata (Cooper et al. 2023). The Australian National Species List databases used by Hespi are the Australian Plant Name Index (APNI 2024), the Australian Bryophyte Name Index (ABNI 2025), the Australian Fungi Name Index (AFNI 2024), the Australian Lichen Name Index (ALNI 2024), and the Australian Algae Name Index (AANI 2025).

The closest match to a taxonomic name in the reference data sets, on the basis of the Gestalt (Ratcliff/Obershelp) approach (Ratcliff and Metzener 1988), is assigned to that field if it is above a certain threshold. Taxonomic names are matched to those in WFO and the Australian National Species List databases without assessment of their taxonomic status (e.g., published, unpublished, accepted, or illegitimate names and synonyms, as defined by the International Code of Nomenclature for Algae, Fungi, and Plants; Turland et al. 2018, 2024). The default threshold is 80%, and this value is adjustable by the user. In this way, minor differences of the taxon name on the specimen label or the extracted data to those in taxonomic reference data sets are corrected. Such differences may be orthographic variants, incorrect spelling of the taxon name on the primary specimen label, the use of nonstandard abbreviation or format in the taxonomic authority, or incorrect text recognition. The closeness of the matches indicates to Hespi whether to use the output from Tesseract or TrOCR when recording the text of the other fields. If no text recognition method is found to be superior (i.e., they generate the same score, or both scores fall below 80%), then handwritten or mixed labels will use the output from TrOCR and other labels will use output from Tesseract.

### Large language model correction

After the text recognition, the results are passed through a multimodal LLM to correct any errors. By default, Hespi uses OpenAI's gpt-4o model. This can be changed to any other model from OpenAI or Anthropic by specifying the model name. The LLM is prompted with the image of the primary specimen label, the list of the desired fields, the currently accepted text for each field and the outputs from the OCR and HTR engines, and how the text has been adjusted after cross-checking with the relevant data sets. The LLM is requested to output the text for any fields where the accepted text is incorrect. Currently, no examples of this process are provided through the prompt and so Hespi is using the LLM as a zero-shot learner. Hespi could be modified to provide the LLM with examples of images from a particular herbarium and so use the LLM as a few-shot learner that will likely improve the results by clarifying expectations (Elliot and Fortes 2023), including for similar primary specimen labels (Brown et al. 2020). This is left for future experimentation.

### Outputs

**Figure 6.**
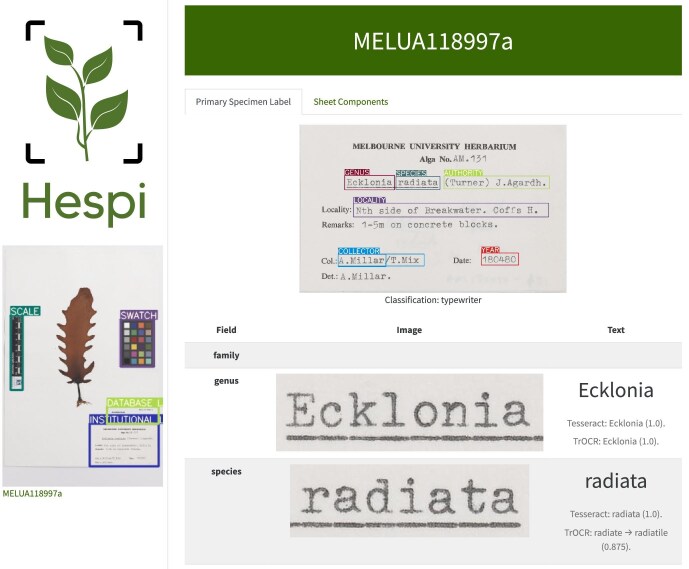
An example screenshot of a Hespi HTML report.

Hespi produces a directory of outputs with the cropped image files and the predictions of both the Tesseract and TrOCR results in both CSV and text files. The pipeline outputs are summarized as an HTML report, which displays the cropped images from each model and the derived recognized text (figure 6). In this way, it is possible to manually cross-check the accuracy of the derived text by comparing it with the original data, visualized from the entire specimen label or the corresponding extracted data field. The CSV file includes the match score between 0 and 1 for the family, genus, specific epithet, and authority, alongside all OCR and HTR results. These scores are a value between 0 and 1, with 1 indicating a perfect match and no corrections made, 0.8 to 1.0 indicating how similar a match was, and 0 indicating no match found with a similarity of 80% or higher.

## Evaluating the end-to-end performance of the pipeline

We created three test data sets for Hespi to evaluate its performance end to end. The first, MELU-T, consists of 100 images of specimen sheets from MELU, where the primary specimen label was either printed or typewritten. The second, MELU-H, consists of 100 images from MELU specimen sheets, where the primary specimen label was handwritten. The final data set, DILLEN, consists of 100 images from the benchmark data set by Dillen and colleagues (2019), including at least 10 specimen sheets from each of the nine institutions represented in that data set. There were no overlaps between the test data sets, and any of the other data sets used for training and validation. Each test data set includes the classification of the type of text on the label and the text for each field on the primary specimen label, which is subsequently referred to as the *ground truth* text. The test data sets and the evaluation script are available online (Turnbull et al. 2024c). The evaluation script shows the accuracy of the label classification and whether or not any particular field should be empty. It also evaluates the similarity between the predicted and the ground truth text for each field. The percentage similarity is measured using the Gestalt (Ratcliff/Obershelp) algorithm. Only fields where text is provided in either the test data set or the predictions are included in the results. If a field is present in either the test data set or the predictions but not the other, then the similarity is zero. All non-ASCII characters and punctuation are removed, and the results are case insensitive. The aggregate results for predictions from Hespi using various components of the pipeline are shown in table 1. This includes results with and without the LLM correction.

**Table 1.** Aggregate results for the three test data sets with text similarity results provided as percentages with and without LLM correction.

			Text similarity no LLM		Text similarity LLM correction	
Test data set	Label classification accuracy	Field present accuracy	Median	Mean	Median	Mean
MELU-T	100.0	98.5%	100.0	91.4%	100.0	92.7%
MELU-H	99.0	97.6%	100.0	81.1%	100.0	88.8%
DILLEN	87.0	84.9%	75.3%	56.8%	100.0	67.1%

**Figure 7.**
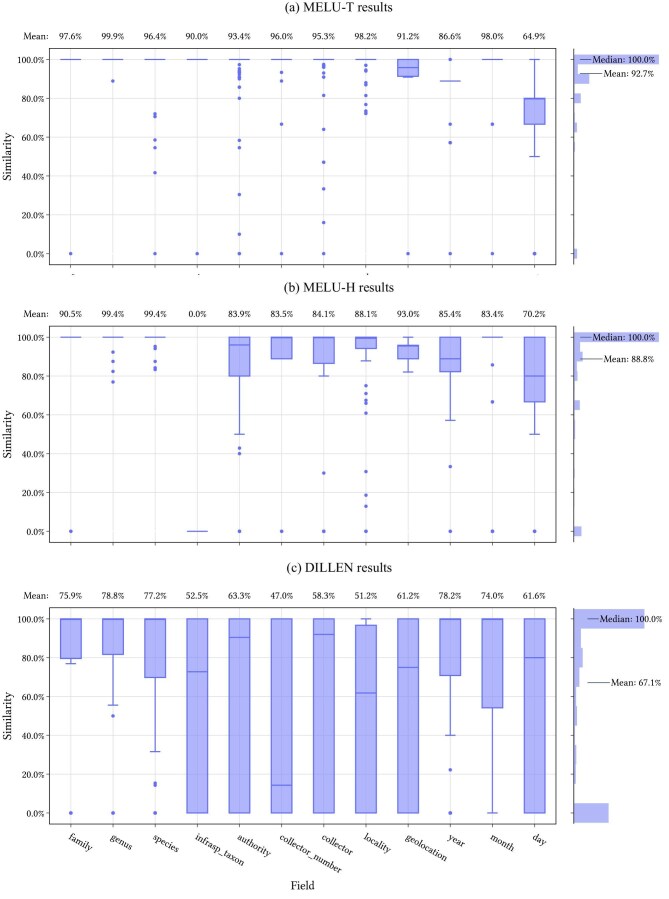
Test results for the full Hespi pipeline, including LLM correction. Box plots showing the quartiles of the text similarity scores for all fields found in the primary specimen labels of the three test data sets. Outliers are shown as points. A histogram of results for all fields in each data set shown on the right.

The distribution of similarity scores for each field type using the full Hespi pipeline is shown in figure 7. Plots of distribution of similarity scores without using an LLM are provided in the supplemental material.

The results for the MELU test data sets were higher for all metrics than those of the DILLEN data set (table 1). This is unsurprising given that a higher proportion of the training data was from MELU and the DILLEN data set is highly diverse in both the layout of the primary specimen labels and in the languages in which the label data are recorded. LLM correction improved the mean similarity score substantially across each data set. Fields with the taxonomic classification of the specimen (i.e., family, genus and specific epithet) are predicted well across the data sets probably because this information is prominent on each label and because of the postprocessing correction of the recognized text from the data set of known entities.

## Innovations and ongoing challenges for specimen data mobilization

In this study, we establish a modular pipeline integrating computer vision and OCR technologies that takes specimen digital image files as input and extracts text data from target fields on the specimen label in a versatile digital file format as output. The pipeline facilitates classification of specimens according to the format of their label data (handwritten, printed or typewritten, or a combination), which enables the scripted collation of specimen sheets with consistent data formats for efficient downstream data handling. Preprocessing of taxonomic identity data is achieved through comparison with taxonomic names lists, with percentage similarity parameters scores reported in the output files. The text is corrected using a multimodal LLM, which markedly improves the results. Extracted text data are parsed into standard Darwin Core fields and can be visually (manually) checked against the label image in an HTML report that is generated.

### Modular AI components

The work presented in the present article achieves significant innovations in the application of deep learning for the digitization of specimen associated biodiversity data: first, object detection of all major nonplant sample sheet components including rulers, color bars, and text data on labels or handwritten on specimen sheets; second, object detection of individual data fields from the primary institutional specimen label; third, the application of specialized HTR software; and fourth, the use of multimodal LLMs for text correction.

The components of the Hespi pipeline are modular, allowing models to be replaced or fine-tuned to specific data distributions for improved performance. Fine-tuned object-detection models (both sheet components and label field) may easily improve results for objects that are not yet accurately detected (e.g., infraspecific taxon data field) or for previously unseen components (e.g., barcodes). As we investigated previously (Thompson et al. 2023a), starting with pretrained model weights and fine-tuning as few as 30 new image annotations was sufficient for accurate bounding box detection of known but previously infrequently encountered sheet components. Instructions for fine-tuning the open-access model weights are available in Hespi's documentation. The particular LLM used for correction can also be replaced by other LLMs as they are developed.

Primary specimen labels are of particular interest for digitization of specimen data and are the text-bearing image file that is typically input into OCR applications (e.g., Barber et al. 2013, Heidorn and Zhang 2013, Guralnick et al. 2024a). The sheet-component model in Hespi produces a set of images of the extracted primary specimen labels, with file names assigned from the input specimens. The Hespi pipeline detects primary specimen labels with high measures of accuracy (mAP50 = 99.3%–99.5%, f1 =  97.7%–98.5%), including for specimens with labels of different formats and with different placement on the specimens. Extracted images of the primary specimen labels are smaller in size, more efficient for file transfer, and can be viewed more efficiently than digital images of entire specimens. Prior work has shown that OCR performance is improved when only text-bearing labels are submitted for processing (Owen et al. 2020). The execution time of OCR processing is also decreased by restricting the input to only text-bearing images (Kirchhoff et al. 2018).

### Sorting of specimen labels

Digital sorting of specimen labels holds the potential to improve efficiency and accuracy of curation workflows by increasing the format consistency within data sets (Cerda and Beach 2010, Tulig et al. 2012, Drinkwater et al. 2014, Takano et al. 2024). For example, Drinkwater and colleagues (2014) noted that more rapid downstream manual data capture and quality control were achieved when specimen sheets were sorted by the country from which specimens were collected or by the collector's name. Collation of similar specimen sheets is enabled in the Hespi pipeline in multiple ways. The label classifier enables segregation of labels according to their print type, enabling downstream curation according to requirements of those data types. In addition, the Hespi pipeline enables rapid and accurate detection of other text-bearing label types (e.g., annotation labels and data written directly on the specimen sheet) and other sheet components (e.g., rulers and color bars) enabling sorting of these specimens, for efficient downstream curation of a set of specimens with consistent data or data formats. Future versions of Hespi will allow for the recognition of text on these components as well.

The diversity of formats of specimen labels within a single institution over time and, among herbaria nationally and internationally, has provided a major challenge for automated digitization of specimen data (Tulig et al. 2012, Owen et al. 2020, Guralnick et al. 2024a). The Hespi pipeline has consistently detected 11 of the 12 target data fields on labels of herbarium specimens drawn from ten international herbaria, which collectively display significant label format diversity. Targeted data fields on the primary specimen labels were consistently and accurately detected (mAP50 = 90.0%–97.8%, f1 = 85.9%–95.9%), with the exception of the infraspecific taxon field. The infraspecific taxon data field was poorly predicted in these models (mAP50 = 68.5%, f1 = 68.1%), presumably because of the rarity of this field appearing in the training data.

### Detection of textual fields

Accurate parsing of textual data extracted from entire specimen labels into Darwin Core fields is a significant challenge (Haston et al. 2017) and represents an impediment to full automation of the post image capture process. By detecting individual data fields within the primary specimen label, Hespi reduces the need for downstream parsing of textual data.

Taxon name fields (family, genus, and specific epithet) were consistently accurately detected (mAP50 = 96.4%–96.7%, f1 = 94.1%–95.3%). These results are comparable with those extracted by Quaesitor (Little 2020), which achieved 0.80–0.97 recall and a 0.69–0.84 precision in the detection of Latin scientific names in the 16 most common languages for biodiversity articles. The detection of infraspecific taxon names was low (mAP50 = 68.5%, f1 = 68.1%), a challenge that was also noted by Little (2020), who noted that infraspecific rank was difficult to discern from hybrid combinations. Milleville and colleagues (2023) applied Google Cloud Vision API to test accuracy of taxon name recognition from 50 specimen labels, noting that only 24% of the words in taxon names were recognized correctly and 36% were partially recognized. Extraction of the taxon authority field (mAP50 = 92.8%, f1 = 90.3%) enabled comparison of extracted taxonomic names with those present in taxon lists during postprocessing. Our postprocessing results indicate that comparisons with global lists of names (e.g., International Plant Names Index), which include both current names and synonyms, increases accuracy of taxon names where global taxonomic diversity is represented.

Numerical date fields were readily detected (mAP50 = 97.5%–97.8%, f1 = 95.4%–95.9%), although the automated extraction of date data risks failing to differentiate the date of specimen collection, determination, or receipt in a collection. Therefore, postprocessing remains necessary to ensure the extracted date data are accurately assigned. Differences in date format in different local contexts, such as the ordering of the day and the month, presents a challenge. The label-field model in Hespi will be biased to Australian conventions because of the training data. This model can be fine-tuned for different contexts. The LLM aspect pipeline has the potential to detect contextual clues and correct incorrect components of the date fields.

Locality data were more challenging, because they were provided as free text and, for many of the specimens in the Dillen data set, were provided in a variety of languages other than English. Georeference data suffered from OCR reading errors—for example, transforming symbols and adding spacing (Guralnick et al. 2024a) that were, in some cases, able to be eliminated through scripted, standardized corrections.

The label-field model prioritizes a subset of fields considered to be authoritative data for the specimen and recognized as essential for most scientific purposes (the minimum information about a digital specimen as per the MIDS digitization standard; Haston and Chapman 2022, MIDS vers. 0.17). For collection curators, the data fields selected hold utility as the core primary collection data fields, to which additional curation data can be added to achieve the expected MIDS level 2 data elements. They also provide baseline data about collection holdings (Cerda and Beach 2010, Tulig et al. 2012) that can subsequently inform targeted curation efforts. For research that relies on specimen associated data, the extracted data fields are fit for purpose for a broad range of downstream research applications (Haston and Hardisty 2020).

The data fields selected were those that are typically present on labels in standardized formats, regardless of the institution from which they were accessioned, which potentially increases the likelihood that they can be accurately detected using these model-based methods. Other free text-based data fields (e.g., habitat, descriptive or collection notes) may be more challenging for bounding-box detection. Those data fields are equally important for extraction to ensure they are available in biodiversity repositories and for subsequent research (Guralnick et al. 2024a).

### Text recognition

The Hespi pipeline includes some postprocessing of the text recognition, to minimize the need for downstream manual processing. One aspect of this is simply formatting text to nomenclatural protocols, so that fields identified as family or genus are capitalized, and those identified as the specific epithet or an infraspecific taxon are set as lower case; another was stripping punctuation from the beginning and end of the family, genus, and specific epithet fields.

Another aspect is the cross-referencing of text identified as family, genus, specific epithet, or authority against a list of each, developed from the Australian National Species List and the World Flora Online Taxonomic Backbone. Initially these reference lists were derived from five indices of the Australian National Species List, incorporating vascular plants (angiosperms, pteridophytes and gymnosperms), bryophytes (mosses, hornworts and liverworts), fungi, lichen, and algae. We extended the cross-referencing ability beyond Australian species by adding taxonomic information from the World Flora Online, which includes vascular plants (angiosperms, pteridophytes, and gymnosperms) and bryophytes. At this stage, the larger list that incorporates names of fungi, lichen, and algae that do not occur in Australia has not been incorporated into the Hespi reference lists.

This cross reference acts as an important check of the quality of the transcription by Hespi, as well as aiming to improve the utility for Hespi users. If a match for the transcribed and formatted text is found in the relevant list, no changes are made. If there are minor errors in the transcription, these can be corrected to the family, genus, specific epithet, or authority that most closely matches the transcript output. The reference list used is determined by the label-field detection; that is, text identified as a genus name will only be checked against the genus reference list. Importantly, changes will only be made to the output if there is a similarity of 80% or higher between the detected text and a taxonomic name in the reference lists, set higher than the default cutoff of 60% to limit any possibility of false matches. In addition, users have the ability to change this cutoff to a higher score, as well as turning off this matching process entirely.

An important consideration with changing the output for close matches to the reference data sets is the risk of introducing errors, with Hespi potentially changing a correct OCR result to an incorrect one. This occurred in 1.5% of all results in the three test data sets when comparing the Hespi result with the ground truth data (with only 13 instances in the 839 results). This was shown in the data as instances where the formatted OCR matched with the ground truth data but was not recognized when compared with the reference lists and so was changed. Closer inspection described below shows that the instances of Hespi changing an OCR result to an incorrect name is actually 0.4% (3 instances out of 839).

The vast majority of these results (9 of the 13 instances) occurred with authority fields, which allows for more variation in how names are recorded. The LLM model was able to account for many of these variations and return the same result as the ground truth. Of those that remained, we found that in seven of these nine authority results, the changed result still referred to the same authority, formatted differently. These differences ranged from as simple as spacing differences (*A.S. George* versus *A.S.George*) or, more commonly, the inclusion of initials (*Wright and Ladiges* versus *I.J.Wright and Ladiges* or *Nordensk*. versus *H.Nordensk.*). In the remaining two instances, a different result was returned because of the use of nonstandard abbreviations for the authority on the taxon label which was not matched with the standard abbreviation in the reference list (e.g., *Schur.* instead of *Schauer*, so Hespi returned *Schau.; Gom.* instead of *Gomont*, so Hespi returned *Gomb*).

In a small number of instances (4 of the 13 instances) Hespi's OCR result matched the ground truth data, but the taxon name was changed through use of the reference data sets and LLM. Of these, three changes (two genus and two specific epithet results) corrected a spelling error on the original label: *Ahnfletia torulosa*, a misspelling of *Ahnfeltia*, the Hespi result; *Odontitis verna*, a misspelling of *Odontites*, the Hespi result; and *Solanum sisymbrifolium*, a misspelling of *sisymbriifolium*, the Hespi result.

In a single instance, Hespi's OCR result matched the ground truth data, but the taxon name was incorrectly matched using the reference data sets and LLM. In this instance, the original label had *Photinia serulata*, a misspelling of *serrulata*. Hespi identified this as the second closest match to *serulata*, but returned the incorrect closest match, *sertulata*.

Overall, the use of the reference data sets and the LLM model resulted in 75% of 189 results with a close but not exact match to the reference data sets that were corrected by Hespi, reducing downstream processing load. The remaining 25% did return errors, but all but three of these would have also been errors without this step being included. Cross checking would still be required for these scores, to identify those where the close match returned was not correct, alongside manual correction of those scoring 0. The scoring system can also help prioritize downstream processing needs, with those scoring 1 requiring the least intervention, and those scoring 0 the most.

Hespi ensures that users can see when and where changes have been made, as well as easily identifying where Hespi has not been able to find a match in these reference lists. Changes are indicated in the console when Hespi is run, and the HTML output provided shows the original transcription (both Tesseract and TrOCR) and any changes made, alongside the selected result and an image of the text. In addition, the CSV output shows all OCR results, and the match score for each relevant field. Downstream users can sort the CSV output by these scores, to easily identify those where no changes were made either because a perfect match was found (a match score of 1), or because no close match was found (a match score of 0), as well as the similarity score (between 0.8 and 1.0) when a change has been made.

Typically, as was done in the present article, the accuracy of data extracted using OCR or HTR technology is assessed by comparison against data resulting from human transcription of specimen labels. However, this comparison can be challenging as textual data are often formatted or interpreted during manual transcription and may not be presented in exactly the same form as the textual data that were written on the label (Owen et al. 2020). A standard protocol for measuring accuracy of data extracted using manual and automated techniques would be beneficial. In addition, as new automated data extraction pathways are developed, an automated, efficient protocol for the comparison of data extraction accuracy is required. These assessment tools could also be applied to develop automated postcapture data correction strategies to optimize data quality and increase the rate of generation of high-quality, accurate digital data.

## Conclusions

We present Hespi, an open-source pipeline for automatically extracting labels containing textual data from herbarium specimen sheets and recognizing a subset of textual data from the original primary specimen label. It takes specimen sheet images and outputs a suite of formatted information, including the text written on the primary specimen label, which is typically the target data for specimen digitization. The text is corrected using a multimodal LLM, which substantially improves the results of standard OCR and HTR engines. Hespi achieves accurate results on the test data sets. The various components of the model can be fine-tuned for other herbaria to improve results in other contexts. It can be incorporated into a wider strategy of digitizing specimen sheets and thereby making available the wealth of data that are associated with those specimens.

## Supplementary Material

biaf042_Supplemental_Files

## Data Availability

Hespi is available as open-source software on Github (https://github.com/rbturnbull/hespi) under the Apache 2.0 Open Source License. It installed directly from the Python Package Index (https://pypi.org/project/hespi/). The automated testing as part of the Continuous Integration/Continuous Deployment (CI/CD) pipeline has 100% code coverage. The whole pipeline runs with a single command and instructions for usage are provided in the online documentation (https://rbturnbull.github.io/hespi/). The data underlying this article are available on FigShare at https://doi.org/10.26188/23597013, https://doi.org/10.26188/25302064, https://doi.org/10.26188/25264408, https://doi.org/10.26188/25648902.

## Author Biography

Robert Turnbull (robert.turnbull@unimelb.edu.au), Emily Fitzgerald, and Karen M. Thompson are affiliated with the Melbourne Data Analytics Platform, and Joanne L. Birch is affiliated with the School of BioSciences at the University of Melbourne, in Melbourne, Victoria, Australia.
