# Supplementary material for: Hespi: a pipeline for automatically detecting information from herbarium specimen sheets
Source: Bioscience. 2025 Jul 17;75(8):637–48. doi: 10.1093/biosci/biaf042 (PMC12352312; doi:10.1093/biosci/biaf042)
Supplement: biaf042_Supplemental_Files [file biaf042_supplemental_files.zip › SupplementaryMaterial-June18.pdf]

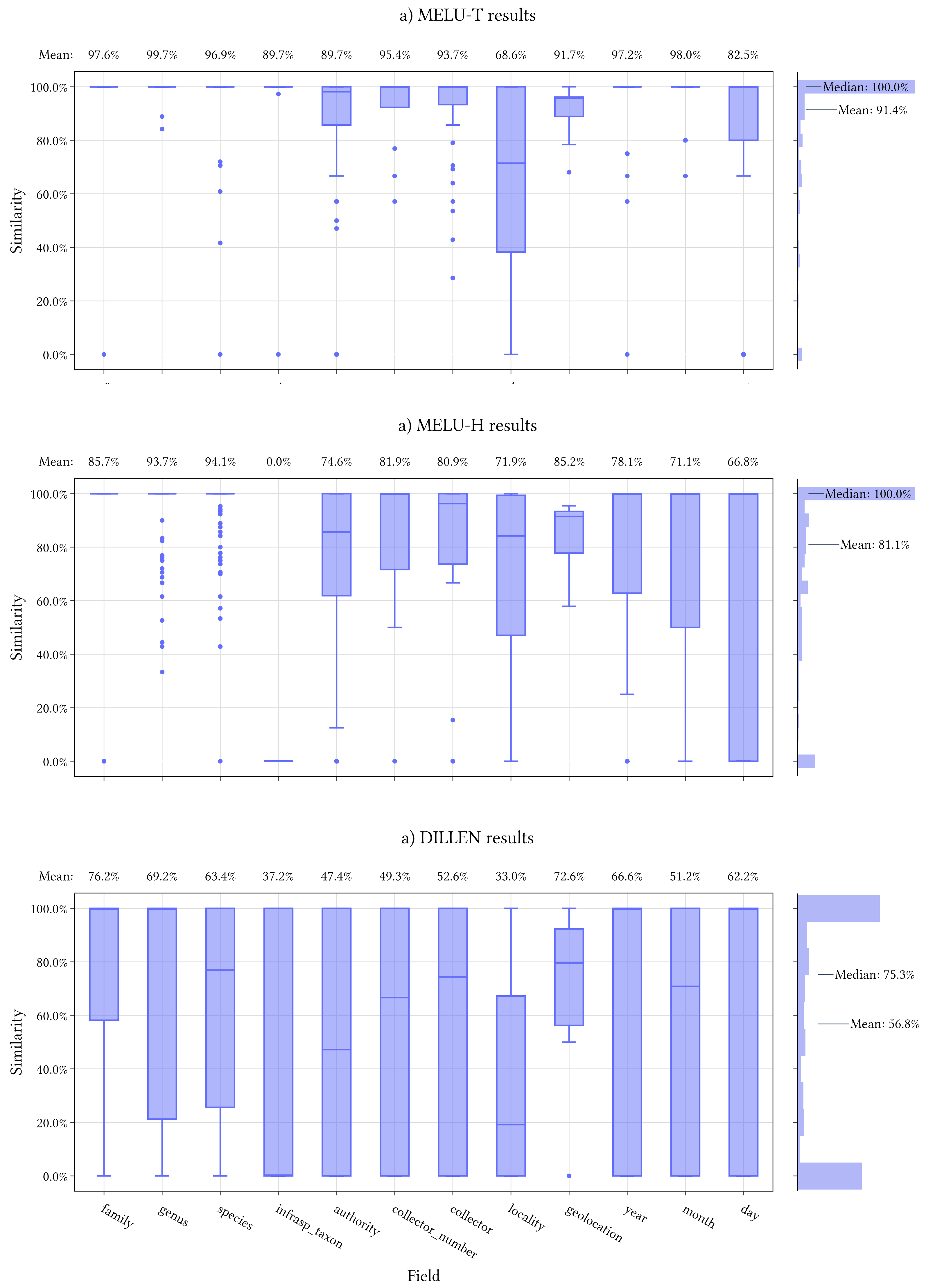

Supplementary Material. Test results without LLM refinement. Box plots showing the quartiles of of the text similarity scores for all fields found in the institutional labels of the three test datasets. Outliers shown as points. A histogram of results for all fields in each dataset shown on right. The results with LLM refinement are available in the main article.
